# Cold-water coral (*Lophelia pertusa*) response to multiple stressors: High temperature affects recovery from short-term pollution exposure

**DOI:** 10.1038/s41598-020-58556-9

**Published:** 2020-02-04

**Authors:** Alexis M. Weinnig, Carlos E. Gómez, Adam Hallaj, Erik E. Cordes

**Affiliations:** 1 Department of Biology, Temple University, Philadelphia, Pennsylvania USA; 2 Departamento de Ciencias Biológicas, Universidad de los Andes, Bogotá, D.C. Colombia

**Keywords:** Biooceanography, Coral reefs, Ocean sciences, Marine biology

## Abstract

There are numerous studies highlighting the impacts of direct and indirect stressors on marine organisms, and multi-stressor studies of their combined effects are an increasing focus of experimental work. *Lophelia pertusa* is a framework-forming cold-water coral that supports numerous ecosystem services in the deep ocean. These corals are threatened by increasing anthropogenic impacts to the deep-sea, such as global ocean change and hydrocarbon extraction. This study implemented two sets of experiments to assess the effects of future conditions (temperature: 8 °C and 12 °C, pH: 7.9 and 7.6) and hydrocarbon exposure (oil, dispersant, oil + dispersant combined) on coral health. Phenotypic response was assessed through three independent observations of diagnostic characteristics that were combined into an average health rating at four points during exposure and recovery. In both experiments, regardless of environmental condition, average health significantly declined during 24-hour exposure to dispersant alone but was not significantly altered in the other treatments. In the early recovery stage (24 hours), polyp health returned to the pre-exposure health state under ambient temperature in all treatments. However, increased temperature resulted in a delay in recovery (72 hours) from dispersant exposure. These experiments provide evidence that global ocean change can affect the resilience of corals to environmental stressors and that exposure to chemical dispersants may pose a greater threat than oil itself.

## Introduction

The combination of human population growth and global industrial development is driving potentially irreversible anthropogenic impacts on the natural world. The current rate and scale of human development is unparalleled, and evidence has shown that human actions are altering the global climate and environment at startling rates^1–4^. Increased atmospheric CO_2_ concentrations contribute to both a decrease in global ocean pH (i.e. ocean acidification) as well as an increase in ocean temperatures^5–7^. Sea surface temperatures have increased on average by 0.6 °C over the past 100 years and models suggest that the deep-sea is warming in some areas at a rate of 0.01–0.1 °C per decade^4,8,9^. The oceans’ surface and bathyal depths (200–4,000 m) are likely to experience increases in temperature of up to 4 °C by 2100^4,10,11^. Furthermore, the ocean’s continuous absorption of excess atmospheric CO_2_ results in a decrease in pH; the surface ocean pH is already 0.1 units lower than preindustrial times and may decline by another 0.3–0.4 units by 2100^12–14^.

In addition to a changing global climate, human activities continue to expose oceanic environments to a wide range of pollutants^15^. Hydrocarbon pollution is one of the most common and widespread forms of marine pollution and can have long-standing effects on marine environments despite standing regulations and monitoring efforts^16^. Upwards of 1.3 million tons of oil is released annually into marine environments; however, roughly 47% of the oil released is from natural seepage^17^. Additionally, as oil exploration/extraction moves farther offshore and into deeper waters, the risk of accidental release increases^18,19^.

The *Deepwater Horizon* (DWH) blowout released at least 518 million liters of crude oil into the deep waters of the Gulf of Mexico (GoM) over a three-month period^20–22^. In response to this oil spill, approximately 7.5 million liters of oil dispersants were applied in an attempt to combat its effects. The purpose of chemical dispersant application is to increase the rate at which the oil is broken down by microorganisms by separating bulk oil into smaller droplets^23,24^. However, there is evidence that dispersants are not as effective at accelerating the biodegradation of hydrocarbons by the microbial community as originally assumed, bringing into question the purpose of their application after an event such as DWH^25^.

**Figure 1:**
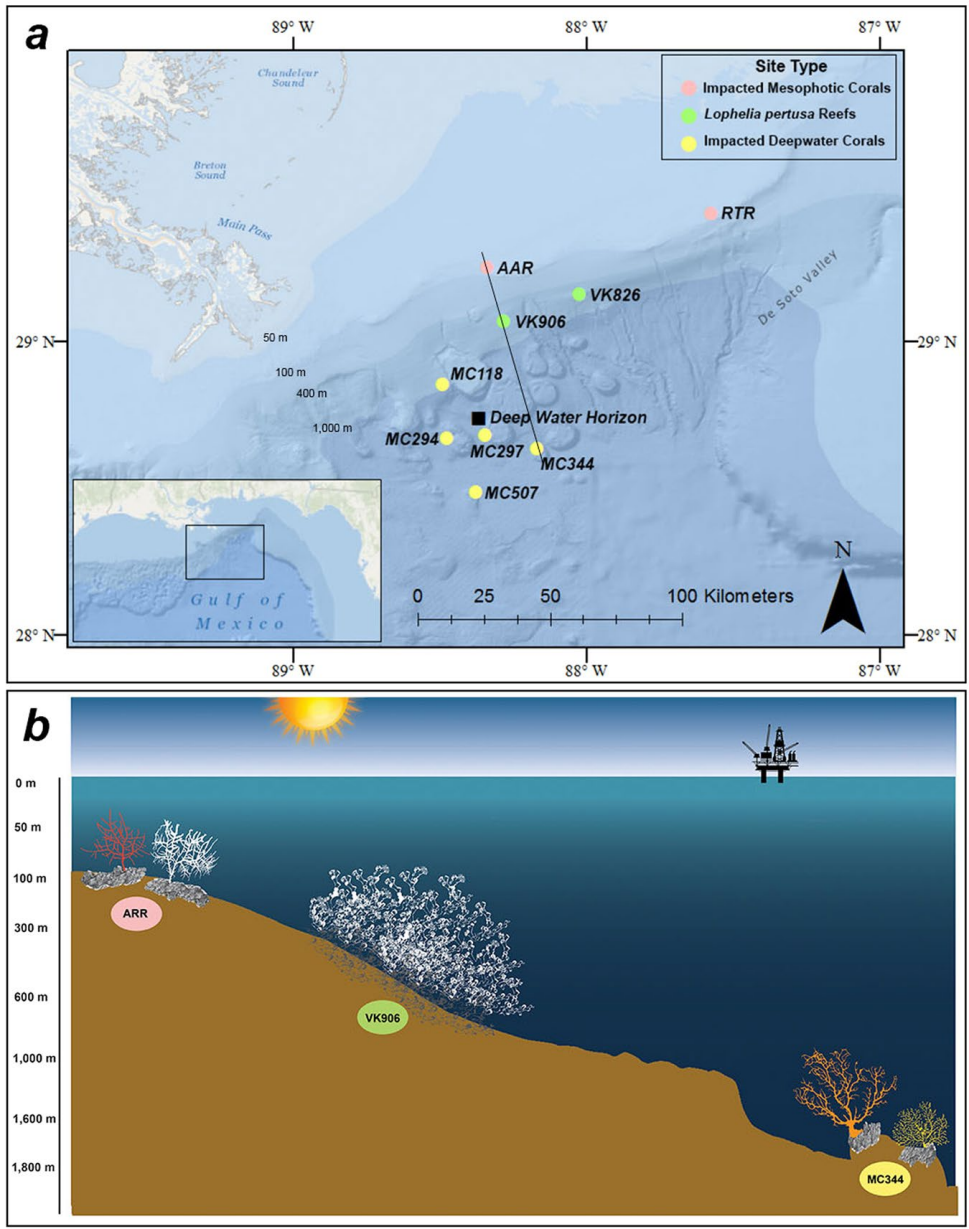
Overview map and a schematic of coral distribution with depth within the Gulf of Mexico. (**a**) Sites of confirmed and potential impact on coral communities in the Gulf of Mexico following the *Deepwater Horizon* (DWH) oil spill (black square). Pink circles indicate mesophotic coral sites impacted by DWH including Alabama Alps Reef (AAR) and Roughtongue Reef (RTR). Green circles represent prominent *Lophelia pertusa* mounds where corals were collected for these experiments including Viosca Knoll 906 and 826 (VK906 and VK826). Yellow circles indicate impacted deep-coral sites after DWH including Mississippi Canyon 118, 294, 297, 334, and 507 (MC118, MC294, MC287, MC334, and MC507). Produced using ArcGIS 10, ESRI (www.arcgis.com). (**b**) A schematic of the cold-water coral communities of the Gulf of Mexico with depth. The depth profile corresponds to the black line in panel “a” drawn through AAR, VK906, and MC344. Examples of corals commonly found at each depth range/site include (from left to right): *Swiftia exserta* and *Hypnogorgia pendula*, at mesophotic sites (ARR), *Lophelia pertusa* mounds (VK906), and *Leiopathes glaberrima* and *Paramuricea biscaya* at deep coral sites (MC344). Note: corals not to scale, enlarged for effect.

One of the most extensive impacts of the DWH incident was on deep-sea ecosystems^26^ (Fig. 1a). Cold-water coral (CWC) communities are an essential component of healthy deep-sea ecosystems and function by contributing to nutrient and carbon cycling, and providing heterogeneous biogenic habitats, feeding grounds, and nurseries for many fishes and invertebrates^27–30^. Within the GoM, CWC communities form a variety of habitats on hard substrata ranging from mesophotic coral assemblages, to upper slope *Lophelia pertusa* reefs, to deeper coral gardens (Fig. 1b). Mesophotic habitats, generally found between 50–200 m, are comprised primarily of black corals and gorgonian octocorals, including *Swifita exserta* and *Hypnogorgia pendula*^31^. While there are scleractinian coral species found at mesophotic depths, the larger *L. pertusa* reef structures are commonly found between 300–600 m in the GoM and form significant biogenic and structural habitat that supports a diverse community of organisms^28,32^. *L. pertusa* larvae initially settle on hard substrate to form individual colonies that then continue to grow and expand both outward and upward, which over geological timescales (millennia and more) can eventually develop large reefs and CWC mounds^33,34^. At greater depths ranging from 600 meters to over 2,000 m, coral gardens, mainly consisting of octocorals and black corals, attach and grow on authigenic carbonates near cold seeps or along the massive Florida Escarpment (Fig. 1b)^35,36^.

There is evidence that oil and dispersant were present in the water column and the surrounding sediments of all three CWC habitat types following DWH^26,37,38^. Shortly after the incident, observations of impacted coral colonies were documented and subsequently studied over time in both the mesophotic and deep coral gardens^37,39–41^ (Fig. 1). While no direct observations of impact were made at the surveyed *L. pertusa* reef sites, there is evidence of oil exposure and the potential that unobserved sub-lethal impacts occurred^26^. Other corals impacted by DWH were found to be covered in brown flocculent material and exhibited signs of stress and mortality, including tissue loss, enlarged sclerites, and excess mucus production^41^. Such obvious physiological stress responses prompted experimental studies to analyze the individual effects of oil, chemical dispersants, and the combination of oil and dispersants on CWC species from GoM deep coral gardens (*Paramuricea biscaya, Callogorgia delta, Leiopathes glaberrima*)^42^ and mesophotic corals (*S. exserta*)^43^. The consensus from these studies was that the dispersants and oil and dispersant mixtures elicited stronger stress responses from the corals than oil exposures alone^42,43^.

In addition to pollution analyses, an increasing number of studies are focusing on CWC responses to changing environmental conditions resulting from climate change including ocean warming, deoxygenation, and acidification. The overall understanding from these studies is that CWC can maintain calcification at low pH levels and are more strongly affected by increasing temperature and low dissolved oxygen concentrations, but that there is substantial individual- and population-level variability in CWC stress responses to these factors^44–50^. While there are numerous studies highlighting the variable effects of climate change and pollution on marine organisms independently, there are very few studies focusing on the potential interactive effects of both climate/ocean change and pollution. To date, the only studies that have attempted to address the interactive effects of oil pollution and climate change have involved coastal microbial and subsurface benthic-dwelling organisms^51,52^. Within these studies, Coelho *et al*. found that the interactive effects of pH and oil pollution can negatively impact the composition and functionality of microbial communities in the sediment, with the potential to intensify the toxicity of oil in marine ecosystems and impair recovery after severe oil contamination events^51,52^. However, these studies focused on estuarine and coastal benthic communities and did not incorporate dispersant exposure into the experimental design.

**Figure 2:**
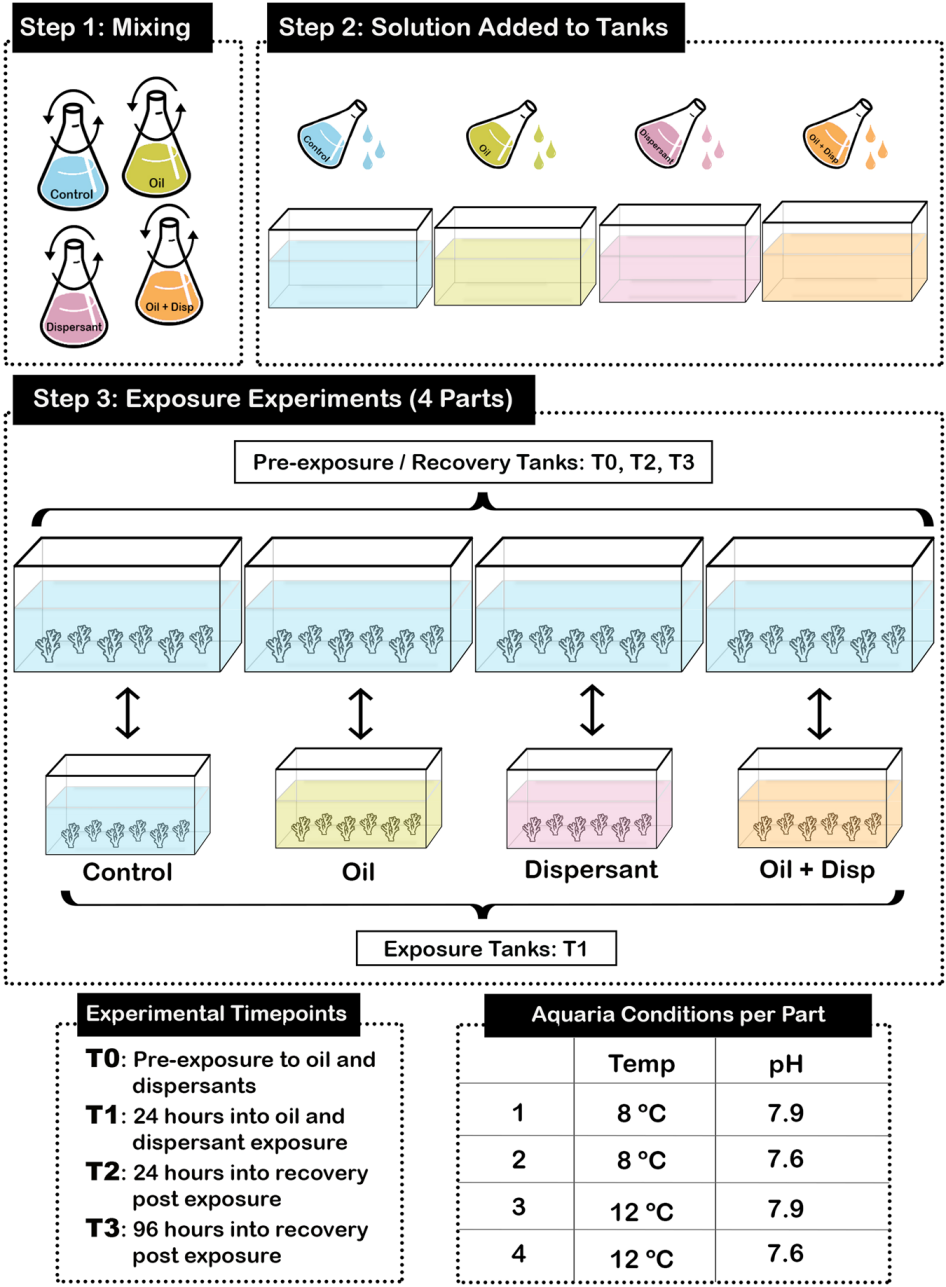
Experimental set up and design. Steps 1 and 2 were performed to prepare the oil, dispersant, and oil + dispersant mixtures before each exposure. Step 3 illustrates the exposure experiments performed for experiments 1 and 2. Health ratings were taken for each coral nubbin at each time point (T0, T1, T2, T3). Experiment 1 consisted of high and low concentrations of oil and dispersant under ambient pH and temperature (7.9 and 8 °C). Experiment 2 consisted of four parts with oil and dispersant exposures run under different combinations of pH and temperature (1: 7.9 and 8 °C; 2: 7.6 and 8 °C; 3: 7.9 and 12 °C; 4: 7.6 and 12 °C). Artificial seawater (ASW) was the control for both experiments.

The present study implemented a set of experiments to investigate the effects of a decrease in seawater pH, increase in seawater temperature, and exposure to oil and dispersants on the health of *L. pertusa* from the GoM (Fig. 2). It is predicted that *L. pertusa* will exhibit a similar stress response compared to other CWC species when exposed to oil and dispersants in that the dispersants will have a more adverse effect than the oil alone. Additionally, as observed in past studies, it is expected that increased temperature will have a greater negative effect on the average health of the coral than lower pH^53^. Also, it is predicted that the combination of increased temperature, decreased pH, and oil/dispersant exposure will produce a synergistic effect. This new information will assist with the prediction of how ocean warming and acidification will affect the ability of *L. pertusa* to respond to future oil spills.

## Results

### Aquaria conditions and carbonate chemistry

Experiment 1 was a pilot experiment to assess the effects of high and low concentrations of oil and dispersant, therefore salinity (35), temperature (8 °C), and pH (7.9) were maintained within a narrow range of the target values. Alkalinity was not directly measured during experiment 1 but all of the in-house artificial seawater has a target alkalinity of 2,300 µmol kg^−1^. During the course of experiment 2, the mean values of temperature, salinity, pH, and alkalinity were maintained within a narrow range of the target values of 8 °C or 12 °C, 35 ppt, 7.6 or 7.9, and 2,300 µmol kg^−1^ (Table 1). These parameters resulted in aragonite saturation (Ω_ar_) values of approximately 1.4, 0.84, 1.59, and 1.06 for the four experimental settings (pH: 7.9 and temp: 8 °C, pH: 7.6 and temp: 8 °C, pH: 7.9 and temp: 12 °C, pH: 7.6 and temp: 12 °C), respectively (please see the *Manipulation of Seawater Chemistry and Temperature* section in Methods).

**Table 1:** Summary of seawater chemistry parameters from each treatment in experiment 2.

Target Experimental Conditions	Salinity	Temp (°C)	A_T_ (µmol kg^−1^)	pH_T_	pCO_2_ (µatm)	HCO_3_^−2^ (µmol kg^−1^)	CO_3_^−^ (µmol kg^−1^)	Ω arag.
pH: 7.9 Temp: 8 °C	35	8.18 ± 0.16	2313 ± 25	7.89 ± 0.06	595.982	2080.02	92.37	1.4
pH: 7.6 Temp: 8 °C	35	8.80 ± 0.60	2298 ± 22	7.64 ± 0.05	1100.48	2158.75	55.19	0.84
pH: 7.9 Temp: 12 °C	35	12.0 ± 0.36	2290 ± 45	7.89 ± 0.04	595.398	2027.82	104.81	1.59
pH: 7.6 Temp: 12 °C	35	12.5 ± 0.38	2319 ± 25	7.68 ± 0.02	1024.82	2145.26	69.56	1.06

Values are given in mean ± SD.

### Exposure effects on *L. pertusa*

#### Experiment 1

**Figure 3:**
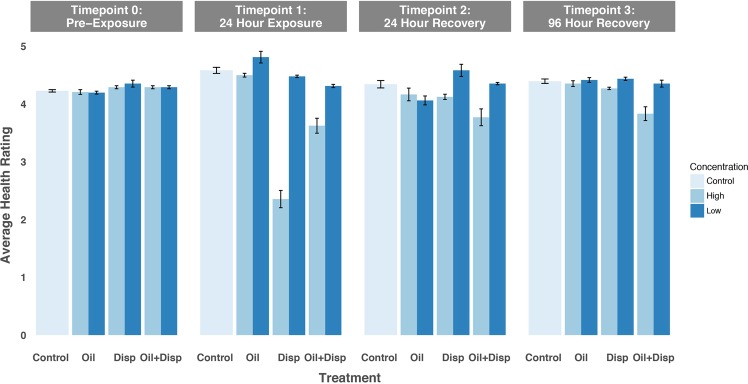
Average health rating for *Lophelia pertusa* fragments over time throughout experiment 1. Fragments were exposed to high and low concentrations of Oil alone, dispersant (Disp) alone, and oil + dispersant (Oil + Disp) mixtures. Artificial seawater (ASW) was used as the Control. Health rating scale 0–5. Bars show standard error.

No mortality was observed in any of the seven treatments (1: oil − high, 2: oil − low, 3: dispersant − high, 4: dispersant − low, 5: oil + dispersant − high, 6: oil + dispersant − low, 7: control) throughout experiment 1. There was no effect of treatment alone on the average health rating across all time points (T0: pre-exposure, T1: 24 hours into exposure, T2: 24 hours into recovery, T3: 96 hours into recovery) [ANOVA *F*_(6,140)_ = 0.63, *P* = 0.7058]. However, there was a significant effect of time point [ANOVA *F*_(3,140)_ = 18.118, *P* < 0.001] and a significant interaction between time point*treatment [ANOVA *F*_(18,140)_ = 16.430, *P* < 0.001] (Fig. 3, Table 2). The post-hoc pairwise comparisons for time point show that the average health ratings at T0 and T3 were significantly different from T1 [Tukey HSD, *P* = 0.0327, *P* = 0.0003]. The recovery time points (T2 and T3) were not significantly different from each other [Tukey HSD, *P* = 0.7404].

**Table 2:** Experiment 1 two-way factorial ANOVA with health rating as the dependent variable showing the effect of timepoint and treatment.

Source of variation	*df*	SS	*F*	P
Treatment	6	0.114	0.63	0.7058
Time point	3	1.644	18.118	**<0.001**
Treatment*Time point	18	8.946	16.43	**<0.001**
Error	140	4.235		

The significant interaction between treatment and time point revealed that the average health rating for the high dispersant concentration treatment at T1 was significantly different from all other treatment*time point combinations [Tukey HSD, P < 0.001 for all 27 comparisons]. For the corals exposed to this treatment, the decrease in average health rating was due mostly to an increase in filament presence and mucus discharge while the nubbins were submerged. However, once the nubbins were returned to normal seawater (T2) the polyps recovered close to the pre-exposure phenotype for the remainder of the experiment. In addition, the average health rating of the corals exposed to the high oil and dispersant treatment exhibited significant differences from the other treatments during the recovery time points (T2 and T3) [Tukey HSD, P < 0.001 for all 37 of the 50 pairwise comparisons]. The corals in the high oil and dispersant concentration also displayed phenotypic changes 24 hours into the exposure (T1). Here, the decrease in average health was due mainly to a decline in tissue health, with portions of the coenenchymal tissue between the polyps detached from the skeleton. In contrast to the recovery observed in the high dispersant concentration treatment between T1 and T2, once the tissue detached from the skeleton it did not recover or reattach when it was returned to normal seawater and the average health rating remained lower than all other treatments for the remainder of the experiment.

#### Experiment 2

**Figure 4:**
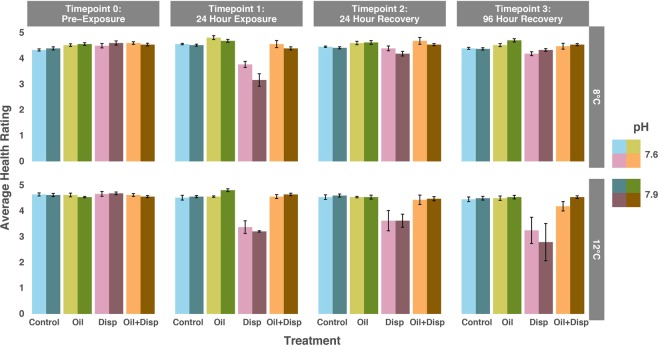
Average health rating for *Lophelia pertusa* fragments over time throughout experiment 2. Fragments were exposed to Oil alone, dispersant (Disp) alone, and oil + dispersant (Oil + Disp). mixtures under different combinations of pH (7.6 and 7.9) and temperature (8 °C and 12 °C). The four combinations of pH and temperature are 1: 7.9 and 8 °C; 2: 7.6 and 8 °C; 3: 7.9 and 12 °C; 4: 7.6 and 12 °C. Artificial seawater (ASW) was used as the Control. Health rating scale 0–5. Bars show standard error.

In the initial phase of the experiment (T0), no mortality was observed in any of the ambient temperature treatments (pH: 7.9 and temp: 8 °C, pH: 7.6 and temp: 8 °C); however, in both of the high temperature treatments (pH: 7.9 and temp: 12 °C, pH: 7.6 and temp: 12 °C) one nubbin died after exposure to the dispersant treatment. There was no difference among the average health ratings across the four treatments at T0 (pre-exposure) for any of the factors (pH, temperature, treatment) as well as their interactions, indicating that the corals were all at similar health states after the acclimation period and prior to the exposures (Fig. 4, Table 3).

**Table 3:** Experiment 2 three-way factorial ANOVA for each timepoint with health rating as the dependent variable showing the effect of pH, temperature, and treatment.

Source of variation	*df*	SS	*F*	P
**Time point 0: Pre-exposure**				
pH	1	0.012	0.533	0.467
Temperature	1	6.83E-35	0.000	1.000
Treatment	3	0.038	0.583	0.628
pH*Temperature	1	0.001	0.029	0.864
pH*Treatment	3	0.095	1.442	0.237
Temperature*Treatment	3	0.023	0.356	0.785
pH*Temperature*Treatment	3	0.076	1.153	0.333
Error	80	1.758		
**Time point 1: 24** **hours into exposure**				
pH	1	0.012	0.175	0.676
Temperature	1	0.033	0.486	0.487
Treatment	3	6.481	0.000	**<0.0001**
pH*Temperature	1	0.032	0.486	0.492
pH*Treatment	3	0.121	0.603	0.615
Temperature*Treatment	3	0.199	0.992	0.401
pH*Temperature*Treatment	3	0.095	0.473	0.702
Error	80	5.354		
**Time point 2: 24 hours into recovery**				
pH	1	0.064	0.522	0.472
Temperature	1	0.188	1.534	0.219
Treatment	3	0.319	0.872	0.459
pH*Temperature	1	0.053	0.431	0.513
pH*Treatment	3	0.095	0.259	0.855
Temperature*Treatment	3	1.253	3.416	**0.021**
pH*Temperature*Treatment	3	0.049	0.133	0.940
Error	80	9.779		
**Time point 3: 96** **hours into recovery**				
pH	1	0.012	0.035	0.852
Temperature	1	0.255	0.765	0.385
Treatment	3	0.154	0.154	0.927
pH*Temperature	1	0.128	0.382	0.538
pH*Treatment	3	0.073	0.073	0.974
Temperature*Treatment	3	2.363	2.360	0.078
pH*Temperature*Treatment	3	0.3482	0.482	0.696
Error	80	26.700		

During the 24-hour exposure period (T1) there was a significant effect of treatment [ANOVA *F*_(3,80)_ = 0, *P* = <0.001]. The post-hoc pairwise comparisons reveal that this effect was due to the decrease in the average health rating of the corals exposed to the dispersant treatment in relation to the other three treatments (oil, oil + dispersant, and control) under all pH and temperature combinations [Tukey HSD, P < 0.001 for all 3 comparisons]. The decrease in health rating was due mostly to an increase in filament presence and mucus discharge while the nubbins were submerged in the dispersant treatment. There was no significant effect of the other factors (pH, temperature) or the interactions during the exposures.

Once the corals entered the recovery phase (T2) there was no longer an effect of treatment alone [ANOVA *F*_(3,80)_ = 0.154, *P* = 0.459] but there was a significant effect of the interaction of temperature*treatment [ANOVA *F*_(3,80)_ = 1.253, *P* = 0.021]. The temperature*treatment effect was driven by the interaction between the dispersant treatment and high temperature (12 °C) [Tukey HSD, P < 0.0061 for all 7 comparisons]. This suggests that the corals that were exposed to the dispersant treatment under high temperature experienced inhibited recovery within the first 24 hours, whereas the corals that were exposed to the dispersant treatment under ambient temperature (8 °C) were able to recover close to pre-exposure phenotypic conditions within 24 hours (T2). There was no effect of the other factors (pH, temperature, treatment) or the interactions during the initial recovery.

At the final time point (T3), 96 hours into the recovery period, there was no difference among the average health ratings for any of the factors (pH, temperature, treatment) or the interactions (Fig. 4, Table 3). The average health rating of the corals that were exposed to the dispersant treatment at elevated temperature (12 °C) was still lower than the other treatments at T3, however the temperature*treatment interaction was not significant due to high variability in the recovery response [ANOVA *F*_(3,80)_ = 2.360, *P* = 0.078].

## Discussion

*Lophelia pertusa* exhibited a decline in health when exposed to high concentrations of dispersant compared to oil + dispersant mixtures or the oil-only treatment, regardless of pH and temperature conditions. In addition, coral fragments that experienced elevated seawater temperature showed slow recovery from dispersant exposure as compared to the fragments in ambient temperature seawater, which recovered their normal phenotype within 24 hours. These experimental data provide important insights into the potential toxicological impacts of oil and dispersant exposure under current and changing ocean conditions on an ecologically significant CWC species. While visible impacts to *L. pertusa* colonies were not observed after the DWH oil spill, there is evidence that some of the reefs were exposed to oil and possibly dispersants, and unobserved sub-lethal effects on these communities remains a possibility^26^.

When considering the components and concentrations of the oil and dispersant mixtures, the hydrocarbon concentrations reported here likely overestimate the actual exposure concentrations given the complexity of crude oil, which is a composite of thousands of compounds with various hydrophilic and hydrophobic properties^54,55^. Additionally, dispersants are composed of an assortment of polar and non-polar surfactants and solvents^56^. It is very likely that some portion of both the oil and dispersant adhered to the flask during mixing and in the experimental aquaria. Furthermore, there may have been loss of water-accommodated oil fractions from volatilization during aeration, coalescence, and/or microbial degradation^57^. Therefore, it is difficult to resolve the exact concentrations of oil and dispersant that each coral polyp encountered at any given time during the exposures. However, if actual exposures were lower than target values, our results are conservative estimates of the effects of oil, dispersant, and oil-dispersant mixtures on *L. pertusa*. In addition, the oil and dispersant exposures under ambient seawater conditions were replicated with consistent health-rating results throughout the separate experimental trials.

One of the primary goals of this study was to better understand *L. pertusa*’s sensitivity to varying concentrations of oil and dispersant that CWCs might experience during an oil spill, based on measurements taken following the DWH spill^38^. Our results show that corals that were exposed to the low concentrations of oil, dispersant, and the combination of the two during experiment 1 did not exhibit a decrease in overall health at any time point (Fig. 3). However, it is important to recognize that reproducing exact conditions experienced by coral colonies during an oil spill is challenging and involves multiple complex phases. Nevertheless, other experimental exposures of CWCs to oil and dispersants also found that as concentration increased, more adverse effects were observed^42^.

*Lophelia pertusa* did not show signs of stress when only exposed to oil regardless of pH and temperature. In some cases, the coral polyps appeared more active (via tentacle extension) in the oil-only treatment relative to the controls (Fig. 4; T1). The lack of stress response to oil might be attributed to *L. pertusa’*s common occurrence in the GoM around areas with low concentrations of natural hydrocarbons seeping from the seafloor and on hydrocarbon-derived authigenic carbonates^28^. While evidence suggests that seep-derived nutrition does not significantly contribute to *L. pertusa*’s energy uptake^58^, it is possible that *L. pertusa* has developed a tolerance to the presence of hydrocarbon compounds in the surrounding water.

A more unexpected result was that the coral nubbins showed little response to the oil and dispersant mixtures during experiment 2. Generally, in previous oil and dispersant toxicity exposures the presence of dispersant, either alone or combined with oil, resulted in a negative health response^42,43^. This could be due to the differing concentrations or ratios of dispersant to oil used in other studies. Previous studies of oil and dispersant toxicity on CWCs (*Paramuricea* type B3, *Callogorgia delta*, and *Leiopathes glaberrima*) from the Gulf of Mexico tested chemically-enhanced water accommodated fractions (CEWAF) with a ratio of 10% of the amount of dispersant to oil^42^, in comparison to the 1.5% of dispersant to oil in the current study. The target dispersant concentration (1.5%) was selected for this study based on the estimation that approximately 500 million liters of oil and 7.5 million liters of dispersant were released during the DWH oil spill, or 1.5% volume of dispersant to total oil^41^. The difference in ratio of oil to dispersant is likely a factor contributing to the various health responses of CWCs to oil and dispersant mixtures.

Within the treatments that induced stress responses, there was variability in recovery response between the nubbins. Some nubbins recovered close to pre-exposure condition while other nubbins died by the end of the experiment and many were in between these two extremes. While this could be because the nubbins were collected at distinct reef patches around Vioska Knoll it is likely that gene flow between these two sites is high given that *L. pertusa* populations in this area of the GOM are considered panmictic^59^. However, previous genotyping efforts indicate that corals collected in this manner represent distinct ‘genets’ with potentially different physiological adaptations^48,53^. There is evidence for genotypic variation in coral stress response, both at the phenotypic and molecular level^48,60,61^. When exposed to low pH (7.6) for short (2-week) or long (6-month) periods of time, some genets of *L. pertusa* were able to continue to calcify, while other genets experienced a net dissolution over the same timeframe^48^. When the same *L. pertusa* fragments were analyzed on the transcriptomic level, ‘collection’ (i.e. genet) had the greatest influence on the variance in gene expression patterns observed and each collection had unique up- and down- regulated transcripts, suggesting that these different collections could be utilizing different pathways to achieve the same goal of homeostasis under stress (Glazier *et al*., submitted). Underlying genetic variation may predispose some individuals or populations to better withstand various stressors; however, the ability to cope with one type of stress does not guarantee success when faced with other stressors or interactions between multiple stressors.

Coral health is complex and made up of myriad factors including not only the physiology of the coral itself, but also the microbial communities that live in the coral tissues and mucous. Zanveld *et al*. (2016) showed that the microbial community associated with shallow-water corals from the Great Barrier Reef was destabilized in response to local stressors and that response was intensified under warmer water temperatures. Following the DWH oil spill, the microbial community of the deep-water column of the GoM was significantly altered and remained in this altered state after the conclusion of the spill^25,62^. In addition, the microbial community of the floc found on CWCs and in the surrounding sediments following the DWH showed evidence of microbial phylotypes with associated genes capable of oil degradation^63^. While the microbial community and its influence on coral health has been more thoroughly characterized for shallow-water corals, it is likely equally significant for CWCs. There has been little research on the impacts of stressors on the microbiome of CWCs and it is likely that the microbiome of *L. pertusa* is altered by changes in temperature, pH, or the presence of oil and dispersant. Changes in the microbiome could lead to significant physiological consequences for the whole coral holobiont. We did not assess microbial community changes in this study, but this is an exciting area for further research.

This study also examined *L. pertusa*’s capacity to recover after short-term (24 hour) oil and dispersant exposures. Across variations in pH and temperature, the oil and oil + dispersant exposures did not cause a decrease in the average health rating during the exposure (T1) and the average health rating remained consistent throughout the recovery phase (T2 and T3). In contrast, across all temperatures and pH values, the dispersant-only exposure caused a significant decrease in average health rating. Furthermore, the average health ratings of the corals that experienced the dispersant treatment in combination with the increased temperature were still significantly lower than the corals in the other treatments 24 hours into recovery (T2). However, the corals that were exposed to the dispersant treatment under ambient temperature were able to recover to the pre-exposure health state 24 hours into recovery (T2). This indicates that the initial and sustained stress to the coral nubbins from the increased temperature had an effect on their ability to cope with the additional stress brought on by the dispersant.

There is evidence in shallow-water coral ecosystems that heat stress, both in the short and long term, can impact a coral’s ability to cope with other forms of stress and/or disturbance^64–66^. Long-term stress from increased temperature can inhibit or even eliminate the ability of coral colonies or entire ecosystems to recover from disturbance^64^. In addition to potential long-term climate change-induced temperature increases, short-term seasonal increases in temperature can interact with localized stressors such as pollution and negatively impact coral health^66^.

Low pH (7.6) did not appear to have an effect on the overall average health of the corals over the short-term exposures of these experiments. Even though pH and alkalinity are important environmental parameters for coral health, it is possible that adverse effects were not observed because the coral response to low pH does not manifest itself through the factors measured by the average health rating. Coral response to temperature has been documented to elicit macroscopic shifts in phenotype, with corals retracting their tentacles and showing visible signs of strain under thermal stress. This type of visible response has not been observed for changes in pH, even if the coral alters its physiology in an effort to mitigate the changes induced by low pH^47,53^. Additional measurements of coral physiology, growth, and gene expression would be necessary to determine if there was an unobserved sub-lethal stress caused by ocean acidification.

Exposing *L. pertusa* to various combinations of oil and dispersants under different pH and temperature regimes allowed us to assess the potential impact of acute disturbances on a prominent CWC at current and projected future ocean conditions. As anticipated, the dispersant had a more adverse effect on coral health than oil. However, surprisingly the oil and dispersant mixtures did not elicit a strong stress response. While it was predicted that the combination of increased temperature, decreased pH, and oil/dispersant exposure would yield the lowest average health rating, this did not appear to be the case. The pH treatment had little impact on the visible health of the coral nubbins and it is likely that if there were adverse effects due to a decrease in pH they would be detectible at the cellular and molecular level (i.e. calcification fluid and gene expression), versus the macroscopic physiological changes assessed in this study. It was the combination of increased temperature and dispersant exposure that resulted in the lowest health ratings. While assessing the health rating through gross morphology is a reliable metric for observing coral stress response, there are undoubtedly other factors contributing to the overall health of the coral and its response to stress. These include factors such as tradeoffs between various physiological processes, altered patterns of gene expression, and changes in the activity and composition of the microbial community. Future studies should focus on these responses, which require additional tools to reveal their more subtle effects. Regardless of the metrics used, it is apparent from this study that increased temperatures can affect the response of a coral to environmental pollution, and that the dispersants used in the DWH oil spill produced more significant effects on coral health than oil alone. These results should inform the types of responses considered in the future when faced with another accidental release of hydrocarbons in the deep ocean. Furthermore, surveys for impacts to coral colonies should occur during or immediately after exposure to environmental pollutants, and that metrics beyond the simple visual assessment of coral health be developed for future impact assessments.

## Materials and Methods

### Sample collection and colony fragmentation

Live *Lophelia pertusa* colonies were collected in the Gulf of Mexico in September 2016 and June-July 2017 aboard the *DSV Ocean Inspector* (Remotely Operated Vehicle (ROV) *Global Explorer; Oceaneering, Inc.*), *MSV Ocean Intervention II* (ROV *Global Explorer*), and *DSV Ocean Project* (ROV *Comanche;* Sub-Atlantic), respectively. All corals were collected from Viosca Knoll leasing area designated by the U.S. Bureau of Ocean Energy Management (BOEM). Corals were collected from two lease blocks within the Viosca Knoll region, 826 and 906 (VK826 and VK906) in a depth range of 392–483 m (Fig. 1; Table 4). The Viosca Knoll sites contain prominent *L. pertusa* mounds with well documented environmental parameters (temperature, pH, salinity, total alkalinity, and aragonite saturation (Ω_ar_))^48,67,68^, which align with the parameters used for coral maintenance and experimental aquaria here. Collections from six distinct coral colonies were made in 2016 and another set of six collections were made in 2017. Individual colonies were collected at least 10 meters apart from conspecific colonies to increase the likelihood of sampling multiple distinct genotypes^67^. Each collection was contained and transported to the surface within a separate insulated “biobox” attached to the ROV. All live corals were kept separate in natural seawater in a temperature-controlled room while aboard the ship until transported in insulated containers from Port Fourchon, Louisiana to Temple University, Philadelphia, Pennsylvania.

**Table 4:** *Lophelia pertusa* collection sites for experiments.

Collection Number	Site	Latitude	Longitude	Depth (m)	Year
1	VK826	29.1653	−88.0175	482	2016
2	VK826	29.1644	−88.0178	480	2016
3	VK826	29.1628	−88.0250	472	2016
4	VK826	29.1650	−88.0175	475	2016
5	VK906	29.0714	−88.3842	397	2016
6	VK906	29.0717	−88.2844	398	2016
7	VK826	29.1587	−88.0103	483	2017
8	VK826	29.1585	−88.0103	481	2017
9	VK906	29.0703	−88.5428	393	2017
10	VK906	29.0703	−88.3842	392	2017
11	VK906	29.1047	−88.5422	393	2017
12	VK906	29.1056	−88.5422	393	2017

Once at Temple University, all corals were kept in 550-liter recirculating aquarium systems with artificial seawater (ASW) prepared using Instant Ocean® or B-Ionic (EVS) and maintained at a temperature of ~8 °C, salinity of 35 ppt, and a pH of ~7.9^69^. Corals were fed MarineSnow (Two Little Fishes) at least 3 days a week^45,53,69^.

Prior to experimentation the corals were fragmented into nubbins (3–6 polyps) and allowed to acclimate to aquaria conditions for at least two months. Nubbins from collections made in 2016 were used in the first experiment and nubbins from collections made in 2017 were used in the second experiment.

### Experimental design

Two independent sets of experiments were conducted in order to test the response of *L. pertusa* to multiple stressors. Experiment 1 was conducted to test low and high concentrations of oil and dispersants based on concentrations detected after the *Deepwater Horizon* oil spill under ambient seawater conditions (pH: 7.9 and temp: 8 °C)^41^. The oil concentration detected in the brown flocculent material (floc) on the surface of live coral at the site MC294 (Fig. 1a) measured 473.8 mg/L. The total amount of Benzene, Tolulene, and Xylene (BTEX) would be 10.42 mg/L, which would account for the majority of the water-accommodated hydrocarbon fraction. The oil concentration detected in the sediments at MC294 was 9,579 mg/L with a BTEX of 210.74 mg/L. Based off these measurements the target high concentration of the water-accommodated fraction of oil tested was 200 mg/L and the target low concentration was 10 mg/L. Dispersant target concentrations were 1.5% of the high and low oil concentrations. The oil + dispersant mixture is the combination of the oil + dispersant concentrations.

For the target high oil concentration treatment (200 mg/L), 162 mL of surrogate oil was added to 1.34 L ASW and mixed at high speed for 48 hours to produce a stock solution at a target concentration of 2,000 mg/L. The target low oil concentration treatment (10 mg/L) was achieved by adding 54 mL of surrogate oil to 0.946 L ASW and mixed at high speed for 48 hours to produce a stock solution at the target concentration of 1,000 mg/L. A separatory funnel was used to retain only the water-accommodated fraction (WAF) and the insoluble layer was discarded. The high and low oil + dispersant mixtures were prepared using the same volumes of oil (162.345 mL and 54.11 mL), with 2.85 mL and 0.81 mL of Corexit 9500 A added (1.5% total oil) to 1.34 L and 0.946 L ASW, respectively, and mixed at high speed for 48 hours to produce a chemically-enhanced WAF (CEWAF). Again, a separatory funnel was used to separate the insoluble layer from the remaining CEWAF. The dispersant stock solution was prepared by adding 2.85 mL of Corexit 9500 A to 1.425 L of ASW with an initial concentration of 848 mg/L. The stock solutions were used to create the experimental treatments with targeted concentration of 200 mg/L (high oil concentration), 10 mg/L (low oil concentration),153.86 mg/L (high dispersant concentration), 7.69 mg/L (low dispersant concentration), 200 mg/L + 1.5% (high oil + dispersant concentration), and 10 mg/L + 1.5% (low oil + dispersant concentration) by combining the stock solution with the appropriate volumes of ASW.

All solutions were transferred into acid-washed glassware aquaria prior to experimentation. There was an acknowledged and unavoidable loss of hydrocarbons and dispersants at the point of transfer from the stock solution container to the experimental aquaria. Therefore, the oil and dispersant concentrations are reported as targeted concentrations and referred to as “oil”, “dispersant” and “oil + dispersant” in the analyses.

**Figure 5:**
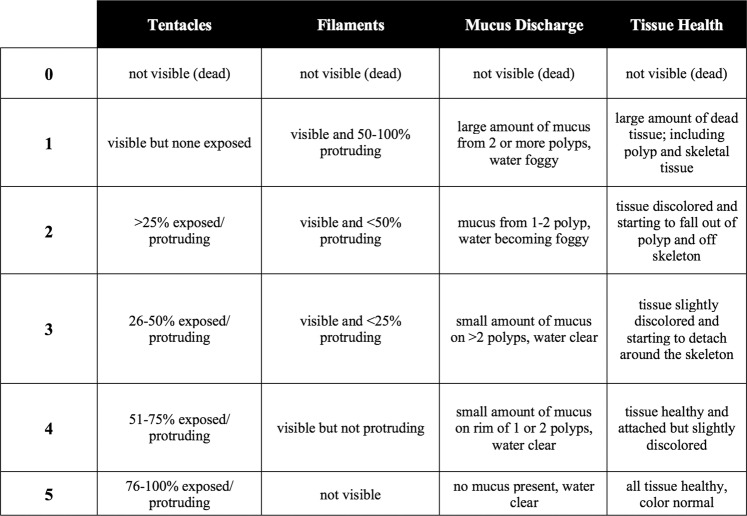
Matrix for assessing the health rating of *Lophelia pertusa*. Each factor (tentacles, filaments, mucus discharge, tissue health) were scored from 0–5, with 0 indicating polyp mortality and 5 indicating signs of optimal health. An average of the four factors was taken for a coral health score for each nubbin at each time point.

For experiment 1, each aquarium (n = 4) had two nubbins from each collection (1–6) for a total of twelve nubbins per aquarium and forty-eight nubbins in experiment 1. Nubbins were acclimated to the conditions of the 56-liter recirculating experimental aquaria (pH: 7.9 and temp: 8 °C) for 48 hours prior to oil and dispersant exposures. One nubbin from each collection was subjected to the treatment exposures (a) oil − high (200 mg/L), (b) oil − low (10 mg/L), (c) dispersant − high (153.86 mg/L), (d) dispersant − low (7.69 mg/L), (e) oil + dispersant − high (200 mg/L + 1.5%), (f) oil + dispersant − low (10 mg/L + 1.5%), and (g) control (ASW only) for 24 hours in individual acid-washed and autoclaved pint-size glass jars before being returned to the larger experimental aquaria for five days. Corals from each treatment type (oil, dispersant, oil + dispersant, control) were kept in separate aquaria to avoid any inadvertent mixing of treatment water. Each sample was photographed (Canon EOS 50D) and a health rating (See Fig. 5 and *Health Ratings* section below) was determined through directly assessing the physiological response by recording tentacle extension, filament state, mucus secretion, and tissue health at four time points (T0, T1, T2, T3).

Experiment 2 was made up of four parts and was conducted by exposing *L. pertusa* nubbins from collections 7–12 to concentrations of oil and dispersant treatments under two different pH (7.6 and 7.9) and temperature (8 °C and 12 °C) conditions. For all four parts of the experiment, each aquarium had one nubbin from each collection (7–12) for a total of six nubbins per aquaria, twenty-four nubbins per part, and ninety-six nubbins for the experiment. The four treatments include (a) surrogate-oil water-accommodated fraction (200 mg/L), (b) Corexit 9500 dispersant (153.86 mg/L), (c) oil + dispersant (200 mg/L + 1.5%), and (d) control (ASW only). No effect was seen in the target low concentration treatments during experiment 1 so only the high concentrations were used in experiment 2. Nubbins were acclimated to the experimental conditions for each part of the experiment; 1: ambient pH and temperature (pH: 7.9 and temp: 8 °C), 2: low pH and ambient temperature (pH: 7.6 and temp: 8 °C), 3: ambient pH and high temperature (pH: 7.9 and temp: 12 °C), and 4: low pH and high temperature (pH: 7.6 and temp: 12 °C) for 48 hours in 56-liter recirculating units. Then the nubbins were subjected to the treatment exposures (a) oil, (b) dispersant, (c) oil + dispersant, and (d) control for 24 hours in an acid-washed 18-liter glass aquarium per treatment before being returned to the larger aquaria for five days (Fig. 2). Each sample was photographed (Canon EOS 50D), a health rating recorded following the same methods as experiment 1, and a genetic sample taken for forthcoming gene expression analyses at the same time intervals as experiment 1 (T0, T1, T2, T3).

### Manipulation of seawater chemistry and temperature

Throughout experiment 2, salinity, temperature, pH, and alkalinity were routinely monitored and recorded. Artificial seawater (B-ionic Seawater System, ESV Aquarium Products Inc) was prepared during the experiments because it provides for accurate manipulation of the components of the carbonate system. The manipulation of seawater pH was achieved by bubbling pure CO_2_ gas into the treatment aquaria using an automated CO_2_ injection system (American Marine Inc, PINPOINT pH Monitor) as in Kurman *et al*.^48^ Total alkalinity (TA) was measured three times during each experiment by acid-titration on an autotitrator (mettle-Toledo DL15, 0.1 mol L^−1^ HCl), with analysis of certified reference materials to confirm accuracy (Dickson Lab, batch #165; Dickson, Sabine, & Christian, 2007; Kurman *et al*.). CO2Calc software^70^ used pH and TA as input variables and the dissolution constants for boric acid and K1 and K2 from Mehrbach *et al*.^71^ refit by Dickson & Millero (1987), KHSO4 from Dickson (1990), and total boron from (Lee *et al*.^72^ to calculate pCO2, HCO3-, CO32-, and Ω_ar_^48,71–75^. Salinity was measured throughout the experiments using a handheld refractometer (Vee Gee 43036), and temperature was recorded every 5 minutes using a temperature logger (onset HOBO Pendant®).

### Health ratings

Each sample was photographed and monitored for signs of stress at the four time points during the experiment. The four main factors contributing to the health rating were tentacle extension, filament extension, mucus discharge, and tissue health (Fig. 5). Tentacle extension is a proxy for how active the polyps are, with a higher proportion of extended and active tentacles corresponding to healthier polyps. Corals have filaments that line the mesenteries inside each polyp, where digestion and gametogenesis take place^76^. When a polyp is under stress it extrudes these mesentery filaments outside the mouth of the polyp, therefore a greater number of filaments visible and protruding corresponds to a lower health rating. Another common stress response for a coral is to increase the amount of mucus discharge to the point where it can produce long mucus trails^77,78^, which corresponds to the lowest health rating in this study. The final factor, tissue health, refers to the discoloration and detachment from the skeleton, indicating a lower health rating. Each factor was scored (0–5) by three independent observers and then the average of all the factors determined the final health rating for each nubbin at each time point.

### Data analysis

Experiment 1 health ratings were averaged for replicate coral nubbins in each treatment and plotted over time to investigate changes in average coral condition. For experiment 2, health ratings were averaged for replicate coral fragments in each experimental condition pair (pH: 7.9 and temp: 8 °C, pH: 7.6 and temp: 8 °C, pH: 7.9 and temp: 12 °C, pH: 7.6 and temp: 12 °C) and treatment (oil, dispersant, oil + dispersant, and control) and plotted over time in R with the ggplot2 package to investigate changes in coral health state. A two-way factorial ANOVA was performed for experiment 1 with health as the continuous variable and treatment and time point as ordinal variables using each individual nubbin as a replicate within each time point. For experiment 2, a three-way factorial ANOVA was performed with health as the continuous variable, and pH, temperature, and treatment as ordinal variables, using each individual nubbin as a replicate within each time point. Tukey’s HSD test was used to test for pairwise differences. All statistical analyses were performed in JMP^79–81^.
